# Comprehensive Analysis of the Expression, Relationship to Immune Infiltration and Prognosis of TIM-1 in Cancer

**DOI:** 10.3389/fonc.2020.01086

**Published:** 2020-09-04

**Authors:** Xiaoxiao Kong, Meili Fu, Xing Niu, Hongxing Jiang

**Affiliations:** ^1^Department of General Surgery, Linyi People's Hospital Affiliated to Shandong University, Linyi, China; ^2^Department of Infectious Diseases, Linyi People's Hospital Affiliated to Shandong University, Linyi, China; ^3^Department of Second Clinical College, Shengjing Hospital Affiliated to China Medical University, Shenyang, China

**Keywords:** cancer, TIM-1, bioinformatics, immune infiltration, biomarker

## Abstract

TIM-1 is a critical gene that regulates T-helper cell development. However, little research has revealed the distribution, prognosis, and immune infiltration of TIM-1 in cancers. TCGA, GEO, Oncomine, TIMER, Kaplan-Meier, PrognoScan, GEPIA, TISIDB, and HPA databases were used to analyze TIM-1 in cancers. High TIM-1 expression was observed in bladder, cholangio, head and neck, colorectal, gastric, kidney, liver, lung adenocarcinoma, skin, uterine corpus endometrial, and pancreatic cancers compared to the normal tissues, and immunofluorescence shows that TIM-1 is mainly localized in vesicles. Simultaneously, high TIM-1 expression was closely related with poorer overall survival in gastric, lung adenocarcinoma, and poorer disease-specific survival in gastric cancer in the TCGA cohort, and was validated in the GEO cohort. Moreover, high expression of TIM-1, correlated with clinical relevance of gastric cancer and lung adenocarcinoma, was associated with tumor-infiltrating lymphocytes in lung adenocarcinoma and gastric cancer. Finally, immunohistochemistry showed TIM-1 expression was higher in lung adenocarcinoma and gastric cancer compared to the normal tissues. In summary, we applied integrated bioinformatics approaches to suggest that TIM-1 can be used as a prognostic biomarker in gastric and lung adenocarcinoma, which might provide a novel direction to explore the pathogenesis of gastric and lung adenocarcinoma.

## Introduction

Cancer is the second leading cause of death worldwide, and the treatment of cancer is still based on traditional surgery, radiotherapy, and chemotherapy (1, 2). With further research done on the molecular mechanism of tumorigenesis and development, research on targeted molecular therapy has made great progress. However, due to the high heterogeneity of tumors, new treatment methods are urgently needed (3). Immunotherapy with immune checkpoint blocking, tumor infiltrating lymphocytes, chimeric antigen receptor T cells, and T cell receptor chimeric T cell have achieved certain effects in the treatment of various tumors. However, only 10–20% of the population can benefit (4, 5). Due to the heterogeneity of tumors, the current biomarkers for predicting prognosis have certain limitations. Therefore, this field requires new biomarkers as prognostic indicators to effectively enhance prognosis and individualized treatment.

TIM protein is a kind of transmembrane glycoprotein expressed on the surface of T cells with similar structural motifs. The human TIM gene family is located in chromatin 5q33.2, and includes TIM- 1, TIM- 3, and TIM-4 (6). TIM-1 protein was first discovered as a receptor of hepatitis A virus in kidney cells of African green monkeys, and plays a role in T-helper cell development (7). TIM-1 is expressed in CD4+ T cells and starts transcription at the initial stage of antigen stimulation, which provides a costimulatory signal for T cell activation, participates in T cell proliferation and differentiation, and inhibits the occurrence of peripheral tolerance (8–10). These findings suggest TIM-1 is a key gene that can regulate T cells and is likely to be an immune marker in cancer.

This study was to analyze the expression and prognosis for TIM-1 and relevance for immune infiltration in cancer. Firstly, we detected the distribution and expression of TIM-1 in human cancer. Secondly, we comprehensively analyzed TIM-1 correlation with prognosis of cancer, which was validated in GEO database. Moreover, we detected the relationship of TIM-1 and tumor-infiltrating lymphocytes (TILs) in cancer. Finally, we used immunohistochemistry to detect the expression of TIM-1 in tumor tissues.

## Methods

### Data Source and Processing

Using the TIMER (Tumor Immune Estimation Resource, https://cistrome.shinyapps.io/timer/) site to analyze the expression of TIM-1 in cancers (11), the mRNA profiling information is from the TCGA (The Cancer Genome Atlas, https://cancergenome.nih.gov/) database. We also use the Oncomine database to analyze the expression of TIM-1 in cancers (12). Bayes test was used to select TIM-1 with a change >=2-fold and a *P*-value cutoff of 0.001 was defined as statistically significant.

### Survival Analysis

GEPIA (Gene Expression Profiling Interactive Analysis, http://gepia.cancer-pku.cn/) site was used to analyze the prognosis of TIM-1 in cancers by using the TCGA dataset (13). PrognoScan database (http://kmplot.com/analysis/) site was used to validate the prognosis of TIM-1 in cancers by using the GEO dataset (https://www.ncbi.nlm.nih.gov/) (14). A univariate Cox *P* < 0.05 was defined as statistically significant. Moreover, we used the Kaplan-Meier plotter database to validate the prognosis of TIM-1 in cancers (15, 16).

### Immune Infiltration

To reveal the immune infiltration of TIM-1 in cancer, we used the TISIDB (tumor-immune system interactions and drugbank, http://cis.hku.hk/TISIDB/index.php) database to infer the relations between abundance of tumor-infiltrating lymphocytes (TILs) and expression of TIM-1. The immune-related signatures of 28 TIL types from Charoentong's study, which can be viewed in the download page. The relative abundance of TILs was inferred by using genomic variation analysis based on gene expression profiles (17).

### Immunofluorescence and Immunohistochemistry

The TIM-1 distribution in cells and expression in cancer were reviewed by using the Human Protein Atlas (HPA, https://www.proteinatlas.org/) (18, 19). The TIM-1 distribution in cells was examined by immunofluorescence, and the protein expression was examined by immunohistochemistry.

### Statistical Analysis

The distribution of TIM-1 in cancer was using HPA site, the expression of TIM-1 in cancer was using the TIMER and Oncomine databases. The survival curve was generated by GEPIA, PrognoScan and KaplanMeier diagrams. The results of KaplanMeier plots, PrognoScan, and GEPIA are displayed with HR and univariate Cox *P*-values from a log-rank test.

## Results

### TIM-1 Expression Profiles in Human Cancer Tissues

**Figure 1:**
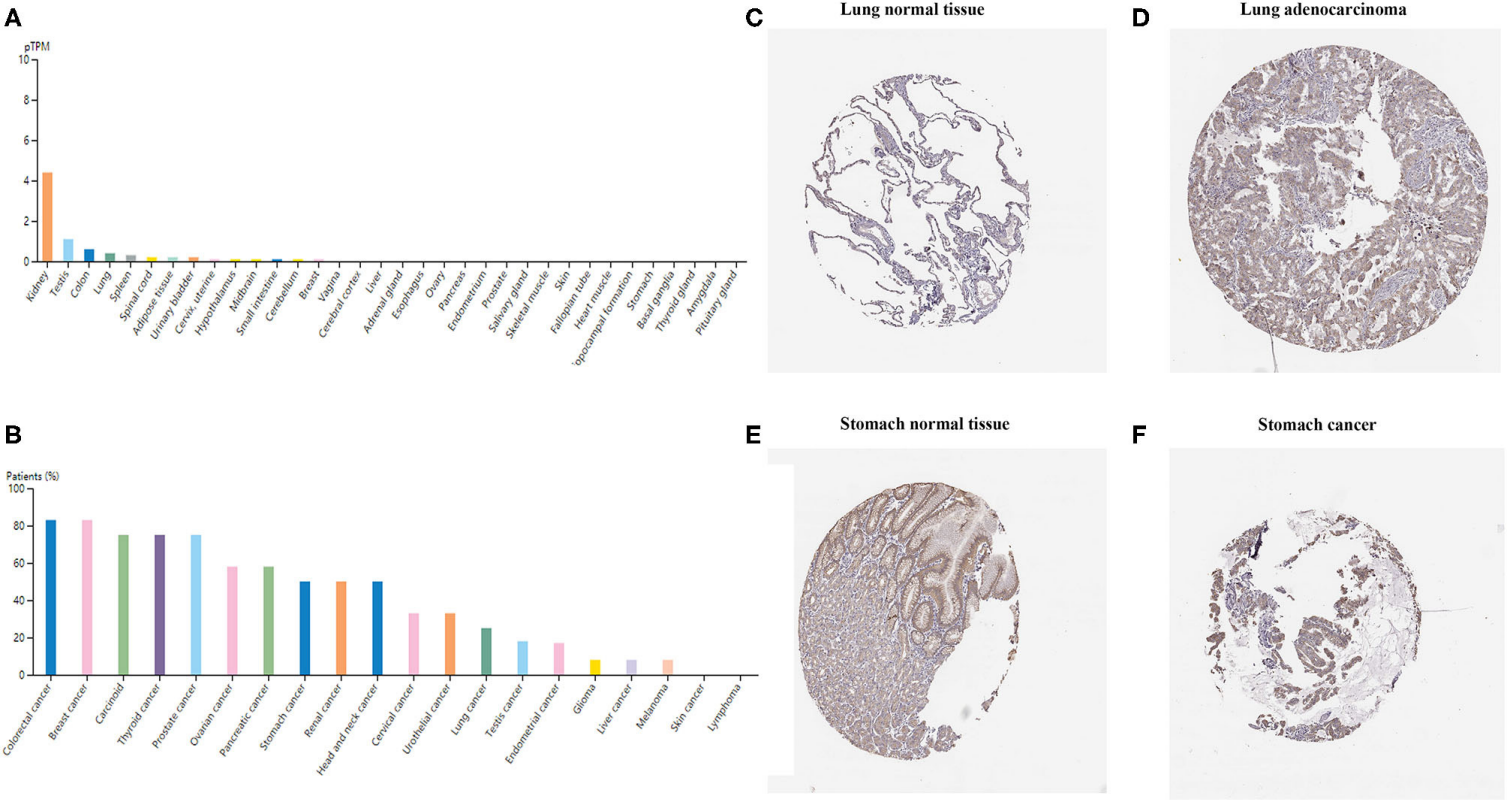
TIM-1 expression profiles in human cancer tissues. **(A)** TIM-1 expression profiles in normal human tissues. **(B)** The protein expression profiles of TIM-1 in human cancer tissues. **(C–F)** Representative IHC images of TIM-1 expression in normal stomach tissues, stomach cancer tissues, normal lung tissues, and lung adenocarcinoma tissues.

**Figure 2:**
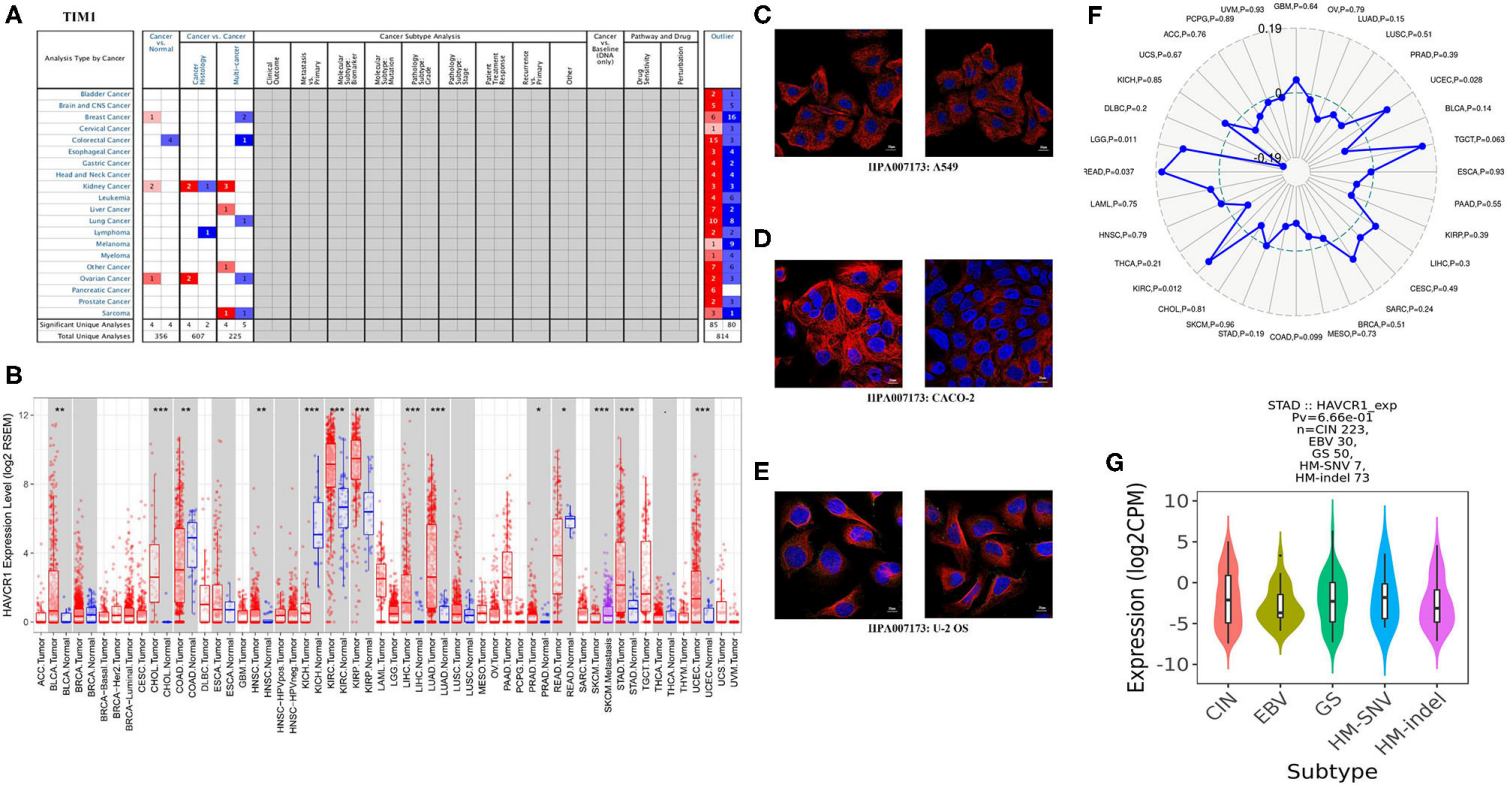
TIM-1 expression levels in different types of human cancers. **(A)** Increased or decreased TIM-1 in datasets of different cancers compared with normal tissues in the Oncomine database. **(B)** TIM-1 expression levels in different tumor types from TCGA database were determined by TIMER (^*^P < 0.05, ^**^P < 0.01, ^***^P < 0.001). **(C–E)** The distribution of TIM-1 in A549 cells, CACO-2 cells, and U-2 OS cells, blue represents nucleus, red represents microtubules, green represents antibody. **(F)** Correlation of TIM-1 expression and MSI in cancers. **(G)** Correlation of TIM-1 expression and molecular subtypes (CIN, EBV, HM-SNV, HM-indel) in STAD.

To examine TIM-1 protein expression in human tumor tissues, the HPA database was used to assess the TIM-1 protein expression in human tumor tissues. As shown in Figure 1A, the TIM-1 mRNA expression was mainly in kidney, testis, and colon in normal human tissues. Then, we detected the TIM-1 protein expression in human tumor tissues by using the GTEx (Genotype-Tissue Expression) database, and TIM-1 protein expression was mainly in colorectal cancer, breast cancer, carcinoid, thyroid cancer, and prostate cancer (Figure 1B). Specifically, immunohistochemistry showed that TIM-1 protein expression was low in glandular cells in normal stomach tissues and normal lung tissues. In comparison, the TIM-1 was higher in expression in stomach cancer tissues and lung adenocarcinoma tissues, and distributed in both cytoplasma and cell membrane (Figures 1C–F). Next, we examined the association between TIM-1 expression and microsatellite instability (MSI). As shown in Figure 2F, higher TIM-1 expression was found in MSI tumors than genomically stable tumors in READ, KIRC, and UCEC in the TCGA dataset (*P* < 0.005). To inspect whether TIM-1 expression was related to the subtype of STAD, we divide STAD into five subtypes (CIN, EBV, HM-SNV, HM-indel). We found high TIM-1 expression had no significant relation to the subtype of STAD (*P* = 0.667) (Figure 2G).

### The Landscape of TIM-1 Expression in Human Cancers

Next, for analysis the different expressions of TIM-1 in tumor and normal tissues, the Oncomine database was used to analyze the TIM-1 mRNA levels in different cancers and normal tissues. As shown in Figure 2A, the TIM-1 expression was higher in breast cancer, kidney cancer, and ovarian cancer compared with the normal tissues. However, the TIM-1 expression was lower in colorectal cancer compared with the normal tissues. To further analyze TIM-1 in tumor and normal tissues, we compared the expression level of TIM-1 in the TCGA dataset. As shown in Figure 2B, compared with normal tissues, the TIM-1 expression was significantly higher in bladder urothelial carcinoma (BLCA), cholangio carcinoma (CHOL), colon adenocarcinoma (COAD), head and Neck squamous cell carcinoma (HNSC), kidney renal clear cell carcinoma (KIRC), kidney renal papillary cell carcinoma (KIRP), liver hepatocellular carcinoma (LIHC), lung adenocarcinoma (LUAD), Prostate adenocarcinoma (PRAD), skin cutaneous melanoma (SKCM), stomach adenocarcinoma (STAD), uterine corpus endometrial carcinoma(UCEC), and rectum adenocarcinoma (READ). However, TIM-1 expression was significantly lower in kidney chromophobe (KICH). In order to investigate the cellular localization of TIM-1 in cancer cells, we used the HPA database to examine the distribution of TIM-1 in cancer cells. As shown in Figures 2C–E, TIM-1 was mainly distributed in vesicles in A549 cells, CACO-2 cells, and U-2 OS cells.

### Survival Analysis of TIM-1 in Cancers

**Figure 3:**
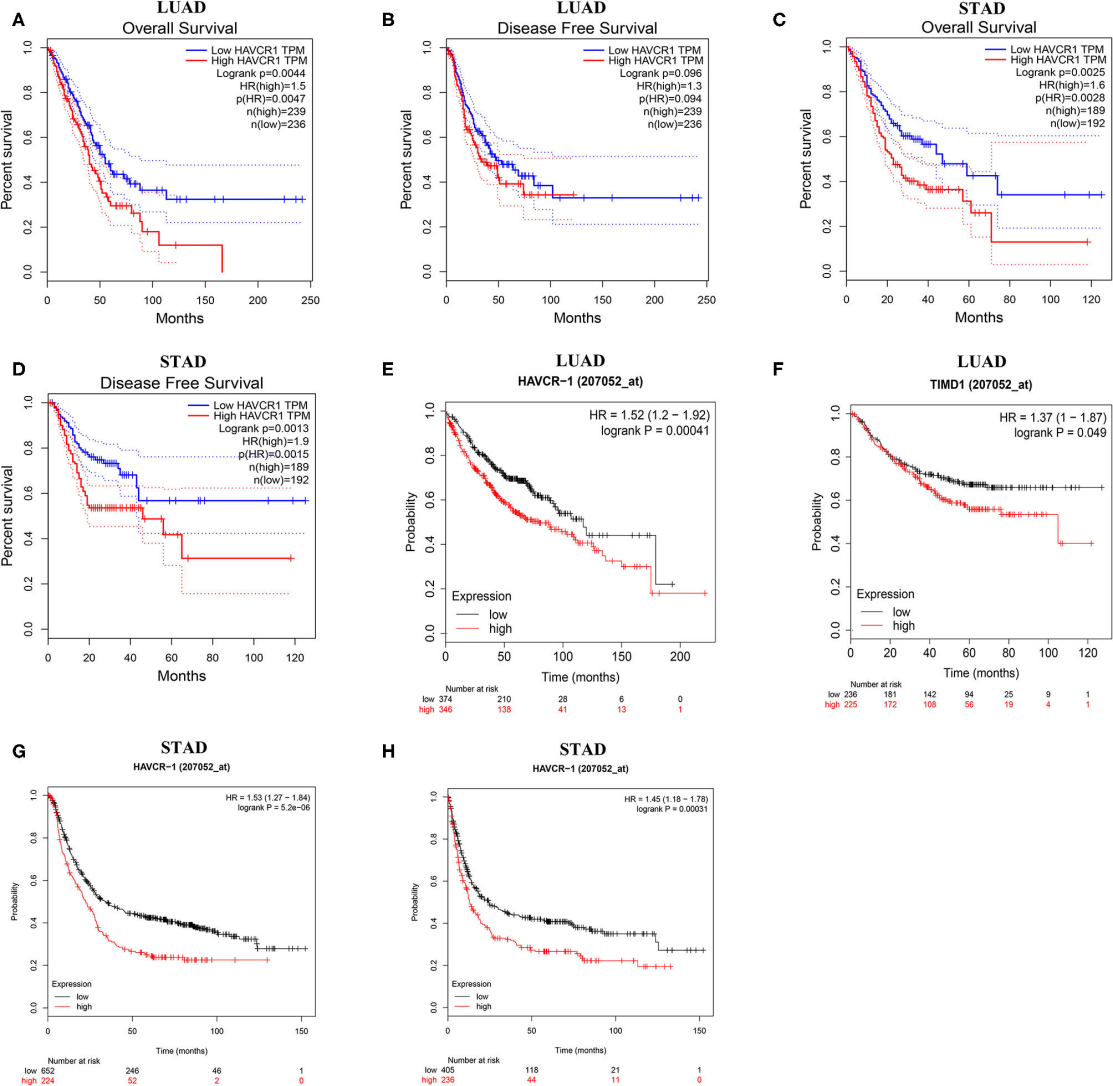
Kaplan-Meier survival curves comparing the high and low expression of TIM-1 in different types of cancer in the TCGA dataset and GEO dataset. **(A–D)** Survival curves of OS and DFS in gastric cancer and lung adenocarcinoma in TCGA cohorts. **(E,F)** Survival curves of OS and PFS in six lung adenocarcinoma cohorts (GSE29013, GSE31210, GSE31908, GSE43580, GSE50081, GSE8894). **(G,H)** Survival curves of OS and PFS in six gastric cancer cohorts (GSE62254, GSE14210, GSE15459, GSE22377, GSE29272, GSE51105).

Next, to inspect whether TIM-1 was related with prognosis in cancer patients, GEPIA site was used to analyze the prognosis of genes in cancers by using the TCGA dataset. Notably, high TIM-1 expression levels were closely related with poorer prognosis of overall survival (OS) and disease-free survival (DFS) in stomach adenocarcinoma, OS in lung adenocarcinoma (Figures 3A–D). Meanwhile, high TIM-1 expression was closely related with better prognosis of DFS in BLCA, KIRC, OS in HNSC, KIRC, SKCM (Supplementary Figure 1).

**Figure 4:**
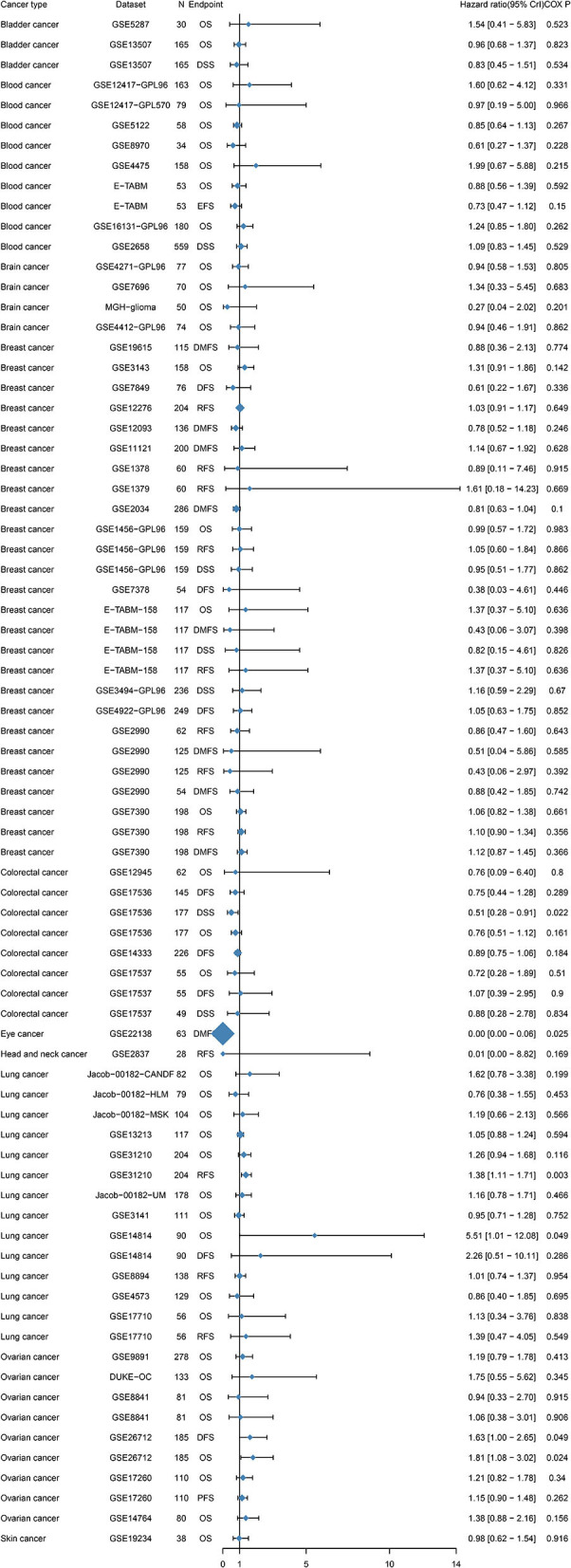
Relation between TIM-1 expression and patient prognosis of different cancers in Prognoscan database.

To further examine the prognostic potential of TIM-1 in cancers, we used the PrognoScan database to examine the TIM-1 in cancers. Three cohorts (GSE14814, GSE31210, GSE26712) included 90 samples, 204 samples and 185 samples in lung adenocarcinoma (20–22) and ovarian cancer, and showed that high TIM-1 expression was closely related with poorer prognosis (lung adenocarcinoma, OS HR = 5.51, 95% CI = 1.01–12.08, Cox *P* = 0.049; DFS HR = 1.38, 95% CI = 1.11–1.71, Cox *P* = 0.003; ovarian cancer, OS HR = 1.81, 95% CI = 1.08–3.02, Cox *P* = 0.024; DFS HR = 1.63, 95% CI = 1.00–2.65, Cox *P* = 0.049). Moreover, two cohorts (GSE17536, GSE22138) included 177 samples and 63 samples in colorectal cancer (23) and eye cancer (24), and indicated that high TIM-1 expression was closely related with better prognosis (colorectal cancer, DSS HR = 0.51, 95% CI = 0.28–0.91, Cox *P* = 0.022; eye cancer, DMFS HR = 0.00, 95% CI = 0.00–0.06, Cox *P* = 0.025) (Figure 4).

To validate the prognostic potential of TIM-1 in stomach adenocarcinoma and lung adenocarcinoma, we used the Kaplan-Meier plotter database to validate the prognostic potential of TIM-1 in stomach adenocarcinoma and lung adenocarcinoma. Interestingly, six cohorts [GSE14210 (25), GSE15459 (26), GSE22377 (27), GSE29272 (28), GSE51105 (29), GSE62254 (30)] included 882 samples in stomach adenocarcinoma and indicated that high TIM-1 expression was closely related with poorer prognosis (OS HR = 1.53, 95% CI = 1.27–1.84, *P* < 0.001; PFS HR = 1.45, 95% CI = 1.18–1.78, *P* < 0.001) (Figures 3G,H). Moreover, six cohorts [GSE29013 (31), GSE31210 (32), GSE31908 (33), GSE43580 (34), GSE50081 (35), GSE8894 (36)], which included 866 samples in lung adenocarcinoma and showed that high TIM-1 expression was closely related with poorer prognosis (OS HR = 1.52, 95% CI = 1.20–1.92, *P* < 0.001; PFS HR = 1.37, 95% CI = 1.00–1.87, *P* = 0.049) (Figures 3E,F).

### High Expression of TIM-1 Correlates With Clinical Relevance of Stomach Adenocarcinoma and Lung Adenocarcinoma

Next, we examined the association between the TIM-1 expression and the clinical relevance of stomach cancer and lung adenocarcinoma patients. As shown in Table 1, high TIM-1 expression was closely related with poorer prognosis in females (OS HR = 1.93, *P* < 0.001; PFS HR = 1.54, *P* = 0.035) and males (OS HR = 1.54, *P* < 0.001; PFS HR = 1.53, *P* < 0.001). Moreover, high TIM-1 expression was closely related with poorer OS and PFS in stage 2 (OS HR = 1.86, *P* = 0.041; PFS HR = 1.88, *P* = 0.041) and 3 (OS HR = 2.19, *P* < 0.001; PFS HR = 1.97, *P* < 0.001) of stomach cancer patients, and poorer OS in stage 1 (HR = 3.36, *P* = 0.047), but was not related with OS and PFS in stage 4 (OS HR = 1.15, *P* = 0.500; PFS HR = 0.78, *P* = 0.270), and PFS in stage 1 (HR = 2.88, *P* = 0.097). Furthermore, high TIM-1 expression was marginally associated with poorer prognosis in the 4 N categories. In addition, high TIM-1 expression was closely related to poorer prognosis in the lauren classification, moderate differentiation, negative and positive HER-2 status. As shown in Table 2, high TIM-1 expression was closely related to poorer OS in males (HR = 1.53, *P* = 0.011), but was not associated with OS and PFS in females (OS HR = 1.33, *P* = 0.144; PFS HR = 1.33, *P* = 0.221), and PFS males (HR = 1.46, *P* = 0.088). Moreover, high TIM-1 expression was not correlated with poorer prognosis in other clinical characteristics (smoking history, stage, and lymph node metastasis), which may be due to lack of sufficient data to analyze.

**Table 1:** Correlation of TIM1 mRNA expression and clinical prognosis in gastric cancer with different clinicopathological factors by Kaplan-Meier plotter (GSE62254, GSE14210, GSE15459, GSE22377, GSE29272, GSE51105).

**Clinicopathological characteristics**	**Overall survival (*n* = 882)**			**Progression-free survival (*n* = 646)**		
	***N***	**Hazard ratio**	***P***	***N***	**Hazard ratio**	***P***
**Sex**						
Female	244	1.93 (1.34–2.79)	3.4e-05	244	1.54 (1.03–2.30)	0.035
Male	567	1.54 (1.23–1.94)	1.9e-05	567	1.53 (1.21–1.95)	4.1e-05
**Stage**						
1	69	3.36 (0.95–11.88)	0.047	69	2.88 (0.78–10.63)	0.097
2	145	1.86 (1.02–3.39)	0.041	145	1.88 (1.02–3.46)	0.041
3	319	2.19 (1.52–3.13)	1.3e-05	319	1.97 (1.32–2.94)	6.7e-05
4	152	1.15 (0.76–1.74)	0.500	152	0.78 (0.51–1.21)	0.270
**Stage T**						
2	253	1.53 (1.00–2.36)	0.049	239	1.61(1.06–2.43)	0.023
3	208	1.58 (1.10–2.27)	0.013	204	1.57 (1.11–2.23)	0.011
4	39	0.25 (0.10–0.65)	0.002	39	0.26 (0.10–0.69)	0.003
**Stage N**						
0	76	3.26 (0.97–10.99)	0.044	72	3.22 (0.96–10.86)	0.046
1	232	2.03 (1.34–3.07)	6e-04	222	2.09 (1.41–3.09)	1.7e-05
2	129	1.73 (1.10–2.72)	0.016	125	1.65 (1.07–2.54)	0.022
3	76	1.38 (0.78–2.46)	0.270	76	0.77 (0.46–1.30)	0.330
1+2+3	437	1.48 (1.14–1.93)	3.6e-04	423	1.48 (1.14–1.91)	0.003
**Stage M**						
0	459	1.62 (1.22–2.15)	6.8e-05	443	1.64 (1.25–2.15)	3.1e-05
1	58	2.49 (1.26–4.92)	6.6e-04	56	2.49 (1.19–5.21)	0.013
**Lauren classification**						
Intestinal	336	2.29 (1.65–3.19)	3.9e-07	263	1.87 (1.30–2.68)	6.2e-05
Diffuse	248	1.48 (1.05–2.07)	0.024	231	1.48 (1.03–2.13)	0.035
**Differentiation**						
Poor	166	1.41 (0.94–2.11)	0.092	121	0.76 (0.48–1.20)	0.235
Moderate	67	1.80 (0.86–3.75)	0.110	67	1.84 (0.91–3.71)	0.083
**Perforation**						
No	169	1.15 (0.77–1.73)	0.490	169	1.19 (0.75–1.87)	0.459
Yes	4	–	–	4	–	–
**Treatment**						
Surgery alone	393	1.41 (1.06–1.88)	0.019	375	1.40 (1.06–1.85)	0.019
5 FU based adjuvant	158	1.35 (0.92–1.98)	0.130	153	1.46 (0.98–2.19)	0.063
Other adjuvant	80	1.97 (0.81–4.84)	0.130	80	1.95 (0.89–4.29)	0.088
**HER2 status**						
Negative	641	1.36 (1.09–1.70)	0.007	408	1.36 (1.04–1.76)	0.021
Positive	425	1.52 (1.12–2.05)	0.006	233	1.62 (1.15–2.29)	0.006

**Table 2:** Correlation of TIM1 mRNA expression and clinical prognosis in lung adenocarcinoma with different clinicopathological factors by Kaplan-Meier plotter (GSE29013, GSE31210, GSE31908, GSE43580, GSE50081, GSE8894).

**Clinicopathological characteristics**	**Overall survival (*n* = 866)**			**Progression-free survival (*n* = 461)**		
	***N***	**Hazard ratio**	***P***	***N***	**Hazard ratio**	***P***
**Sex**						
Female	318	1.33 (0.91–1.96)	0.144	235	1.33 (0.84–2.09)	0.221
Male	344	1.53 (1.10–2.13)	0.011	226	1.46 (0.94–2.25)	0.088
**Smoking history**						
No	143	1.41 (0.63–3.17)	0.400	143	1.67 (0.90–3.09)	0.101
Yes	246	1.22 (0.77–1.95)	0.397	243	1.38 (0.89–2.14)	0.145
**Stage**						
1	370	1.84 (1.24–2.72)	0.002	283	1.24 (0.77–2.00)	0.374
2	136	1.69 (1.03–2.77)	0.035	103	1.41 (0.81–2.46)	0.221
3	24	0.92 (0.33–2.56)	0.872	10	–	–
4	4	–	–	0	–	–
**Stage T**						
1	123	1.00 (0.52–1.82)	0.999	47	7.15 (0.86–59.53)	0.034
2	105	1.04 (0.60–1.79)	0.898	93	0.98 (0.52–1.83)	0.942
3	4	–	–	2	–	–
**Stage N**						
0	184	0.80 (0.49–1.30)	0.366	102	0.53 (0.24–1.20)	0.121
1	44	1.64 (0.74–3.63)	0.216	38	3.41 (1.22–9.57)	0.013
2	3	–	–	25	–	–
**Stage M**						
0	231	0.95 (0.64–1.41)	0.799	142	1.16 (0.65–2.05)	0.616
1	1	–	–	0	–	–
**Chemotherapy**						
No	21	1.32 (0.32–5.37)	0.699	11	–	–
Yes	36	2.84 (0.74–10.86)	0.112	19	–	–

### TIM-1 Expression Was Correlated With TILs

**Figure 5:**
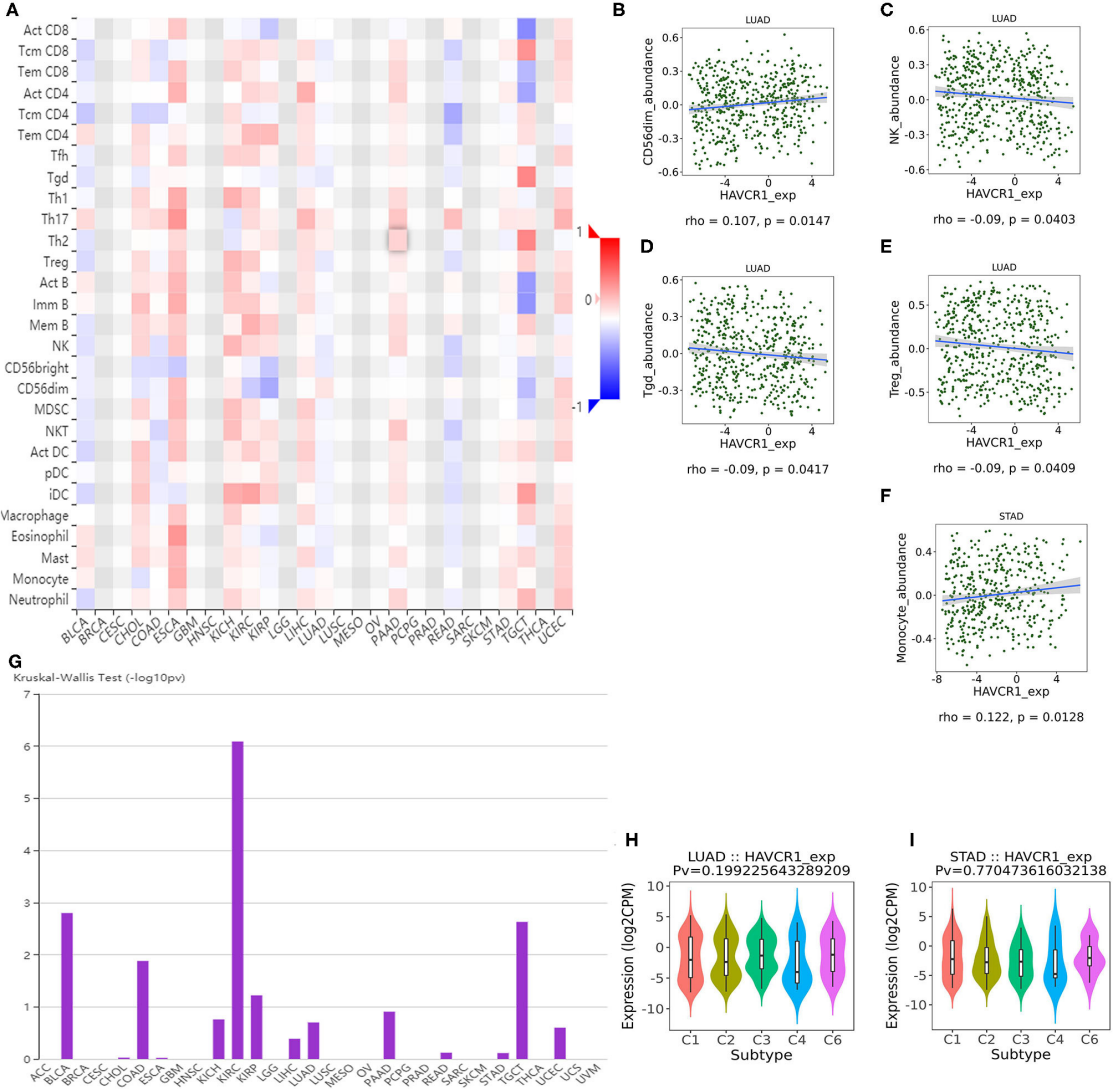
Correlation of TIM-1 expression with immune infiltration level in cancer. **(A)** The landscape of relationship between TIM-1 expression and TILs in different types of cancer (red is positive correlated and blue is negative correlated). **(B–F)** TIM-1 expression was positively closely related with infiltrating levels of CD56dim natural killer cell in lung adenocarcinoma and monocyte in stomach cancer, and was negatively correlated with infiltrating levels of natural killer cell, gamma delta T cell, and regulatory T cell in lung adenocarcinoma. **(G)** The landscape of relationship between TIM-1 expression and immune subtypes across human cancers. **(H,I)** Correlation of TIM-1 expression and immune subtypes (wound healing, IFN-gamma dominant, inflammatory, lymphocyte depleted, TGF-β dominant) in stomach cancer and lung adenocarcinoma.

TILs are an independent predictor in cancers (37, 38). Therefore, the TISIDB database was used to infer the relations between abundance of TILs and expression of TIM-1. The landscape of the relationship between TIM-1 expression and TILs in different types of cancer was shown in Figure 5A. The relations between abundance of 28 TIL types and expression of TIM-1 was weakly to moderately correlated. Specifically, TIM-1 expression was positively closely related with infiltrating levels of CD56dim natural killer cell in lung adenocarcinoma (*r* = 0.107, *P* = 0.015) and monocyte in stomach cancer (*r* = 0.122, *P* = 0.013), and was negatively correlated with infiltrating levels of natural killer cell (*r* = −0.090, *P* = 0.040), gamma delta T cell (*r* = −0.090, *P* = 0.042), and regulatory T cell (*r* = −0.090, *P* = 0.041) in lung adenocarcinoma (Figures 5B–F). Next, we detected the associations between TIM-1 expression and immune subtypes across human cancers, and the landscape of relationship between TIM-1 expression and immune subtypes across human cancers was shown in Figure 5G. Specifically, TIM-1 expression was not correlated with immune subtypes (wound healing, IFN-gamma dominant, inflammatory, lymphocyte depleted, TGF-β dominant) in stomach cancer and lung adenocarcinoma (Figures 5H,I).

## Discussion

Due to advances in treatment, the mortality rate of tumors has been declining in recent years, a large part of which is due to immunotherapy (39). Immunotherapy represented by anti-PD-1/PD-L1 monoclonal antibody drugs and CAR-T cell therapy has attracted much attention, and encouraging results have continued. Both of them are essentially the ability of the human autoimmune system to recruit and activate human core immune guardian-T cells to identify and clear cancer cells through antigen-antibody response (1). However, not every patient responds to this treatment, especially in gastric cancer (40, 41). Therefore, there is an urgent need to clarify and identify new immune-related therapeutic targets. High throughput technology has been widely employed to investigate gene expression in numerous tumors, providing a novel method to identify significant genes and explore tumor progression and initiation.

Here, we report that high TIM-1 expression was observed in bladder, cholangio, head and neck, colorectal, gastric, kidney, liver, lung adenocarcinoma, skin, uterine corpus endometrial, and pancreatic cancers compared to the normal tissues, and immunofluorescence shows that TIM-1 is mainly localized in vesicles. Simultaneously, high TIM-1 expression was closely related with poorer overall survival in gastric, lung adenocarcinoma, and poorer disease-specific survival in gastric cancer in TCGA cohort. High TIM-1 expression was closely related with poorer overall survival in gastric and lung adenocarcinoma, and poorer disease-specific survival in gastric cancer, lung adenocarcinoma was validated in GEO database. Moreover, high expression of TIM-1 correlates with clinical relevance of gastric cancer and lung adenocarcinoma.

TIM-1 has a certain value in evaluating the disease progression and survival prognosis of patients with cancer (42). TIM-1 expression level in non-small-cell lung cancer is significantly correlated with tumor size, degree of differentiation, clinical stage, lymph node, and distant metastasis. Moreover, the overall survival rate of non-small-cell lung cancer patients with high expression of TIM-1 is significantly lower than that of patients with low expression of TIM-1. In cell experiments, it was also found that inhibition of TIM-1 expression could inhibit the migration and invasion ability of A549 and SK-MES-1 cells *in vitro*, respectively (43). All these suggest that TIM-1 plays an important role in the invasion and metastasis of cancers.

We also investigated the relationship between immune infiltration and TIM in cancer, and found TIM-1 was positively associated with tumor-infiltrating lymphocytes of CD56dim natural killer cell in lung adenocarcinoma and monocyte in gastric cancer, and was negatively correlated with infiltrating levels of natural killer cell, gamma delta T cell, and regulatory T cell in lung adenocarcinoma. Finally, immunohistochemistry shows TIM-1 expression was higher in lung adenocarcinoma and gastric cancer compared to the normal tissues. Thus, our study provides us clues to understand the potential role of TIM-1 in tumor immunology and may be a potential prognostic molecular marker. TIM-1 mainly provides stimulating signals for the activation of T cells, participates in the proliferation and differentiation of T cells, and inhibits the occurrence of peripheral tolerance (44). When Th cells differentiate into Th1 and Th2 cells, TIM-1 is only highly expressed on Th2 cells and has been shown to have an important relationship with mouse Th2 cell-mediated airway hyperreactive diseases (45). There is evidence that when T cells are stimulated, the tyrosine residue of TIM-1 protein extending into the cell is phosphorylated, which is also the promoter of IL-4, and an activating nuclear factor of T cells/activator protein-1 (NFAT/AP-1) dependent transcription provides costimulatory signals (46). The regulation of cytokine transcription is controlled by many transcription factors, among which NFAT is one of the most thoroughly studied transcription factors (47). It has been proved that four members of NFAT are expressed in lymphocytes (48). The activity of most members of the NFAT family is regulated by Ca^2+^. When the concentration of Ca^2+^ in the cell increases, dephosphorization mediated by Ca^2+^/calmodulin phosphatase occurs, and NFAT molecules can enter the nucleus to play a role. Furthermore, the activation of NFAT molecule follows the activation of Ca^2+^/calmodulin dependent phosphatase, while in T cells, the level of free Ca^2+^ is higher, so the activity of Ca^2+^/calmodulin dependent phosphatase increases, and NFAT can maintain the activation state in the nucleus for a long time and promote the transcription of some genes. In addition to Ca^2+^/calmodulin dependent phosphatase, there are many proteins and signaling pathways that regulate NFAT, such as T cell receptor (TCR) cross-linking (49–51). Moreover, the activation of Lck and ZAP70 leads to calcium mobilization in T cells, which leads to NFAT-dependent reporter gene expression (52). Experiments have shown that the cells co-expressing TIM-1 and NFAT/AP-1 responded more strongly to the stimulation from TCR/CD3 complex, while the activity of NFAT/AP-1 did not increase, indicating that TIM-1 may play a costimulatory role in NFAT/AP-1-dependent transcription (53).

It has been reported that the use of a TIM-1 specific antibody in a mouse asthma model can inhibit the occurrence and severity of airway hyperreactive inflammation by reducing the production of cytokines such as IL-10 and IL-13, and earlier studies have also mentioned that a positive hepatitis A virus reduces an individual's susceptibility to certain allergic diseases (54). Therefore, it can be inferred that the interaction between TIM-1 and ligand can enhance the activation of T cells and increase the production of Th2 type cytokines, while blocking this interaction can greatly inhibit the activity of Th2 cells, thus regulating the immune response mediated by Th2 cells. Therefore, TIM-1 plays an important role in regulating the differentiation, proliferation, and effector function of Th cells. The action of TIM-1 coding products and ligands can promote the differentiation and proliferation of Th cells and enhance the immunity of Th2 cells (55). Through the study of the TIM-1 gene, we can have a deeper understanding of all kinds of inflammatory responses mediated by Th cells (such as asthma, allergic rhinitis, and other autoimmune diseases) and the mechanism of cancer, so as to have a far-reaching impact on the prevention and treatment of these diseases.

There are some limitations to this study. Firstly, there is no experimental validation of the predicted results, and the authors should pay attention to the experimental validation of the predicted results by different methods to be further confirmed, for example by RT-PCR in addition to high-throughput sequencing. TIM-1 is down-regulated in KICH and a protective effect was detected in colorectal cancer and eye cancer as demonstrated in two cohorts (GSE17536, GSE22138). The included studies did not cover all previous published literatures revolved in TIM-1 and certain cancers with controversial findings. Therefore, experimental validation of the predicted results is still needed by RT-PCR to be further confirmed.

In summary, we applied integrated bioinformatics approaches to suggest that high TIM-1 was closely related with poor prognosis in gastric cancer and lung adenocarcinoma. Therefore, TIM-1 can be used as a prognostic biomarker in gastric cancer and lung adenocarcinoma, which might provide a novel direction to explore the pathogenesis of gastric and lung adenocarcinoma.

## Data Availability Statement

Publicly available datasets were analyzed in this study. This data can be found here: https://cancergenome.nih.gov/.

## Author Contributions

XK and HJ designed the research, analyzed the data, and wrote the paper. MF and XN performed the data analysis and interpreted the data. All authors read and approved the final manuscript.

## Conflict of Interest

The authors declare that the research was conducted in the absence of any commercial or financial relationships that could be construed as a potential conflict of interest.

## Supplementary Material

Supplementary Figure 1 Correlation of TIM-1 expression with prognostic values in diverse types of cancer.
