# Supplementary figures and images for: Comprehensive Analysis of the Expression, Relationship to Immune Infiltration and Prognosis of TIM-1 in Cancer
Source: Front Oncol. 2020 Sep 4;10:1086. doi: 10.3389/fonc.2020.01086 (PMC7498659; doi:10.3389/fonc.2020.01086)

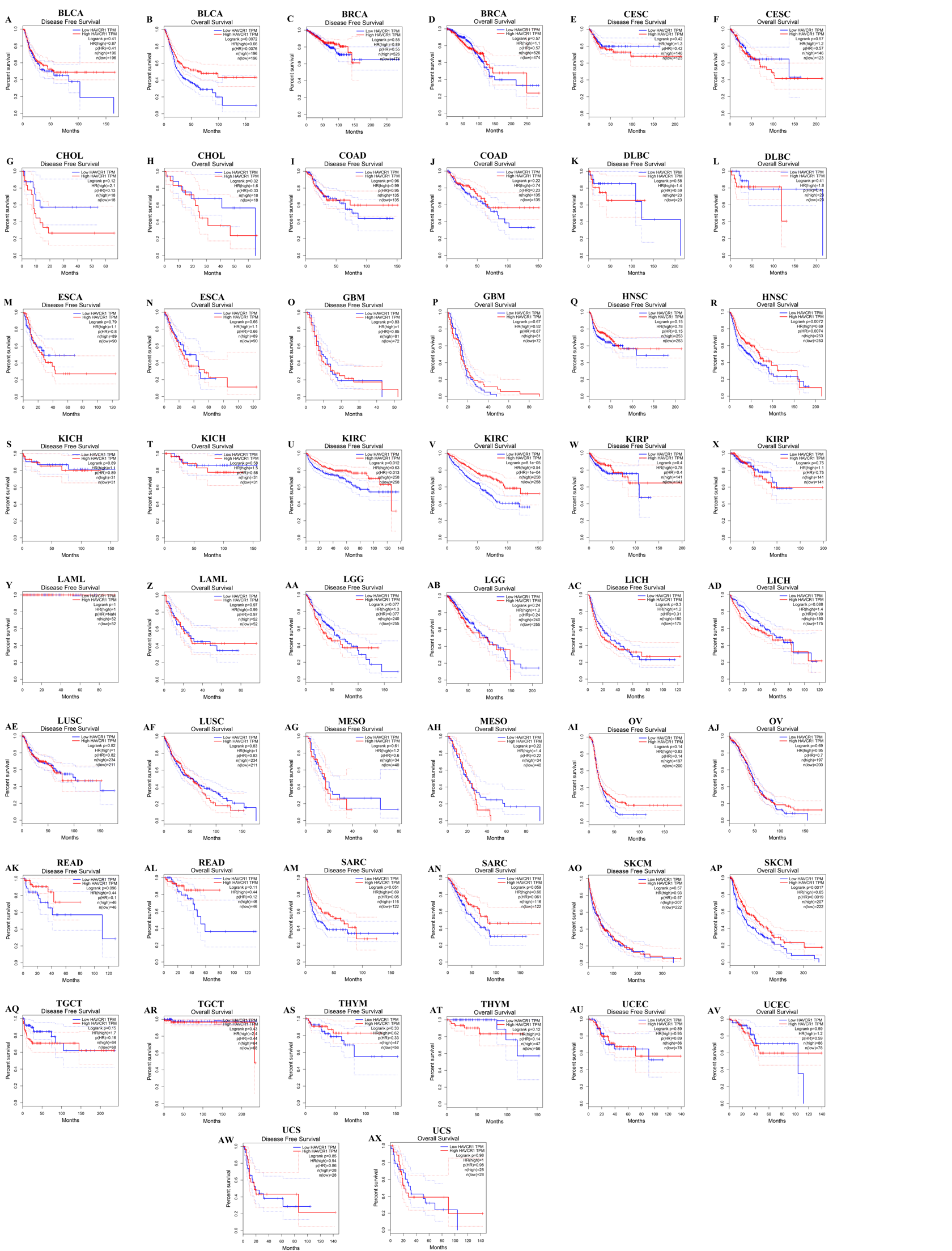

Supplement: Supplementary Figure 1 — Correlation of TIM-1 expression with prognostic values in diverse types of cancer. [file Data_Sheet_1.ZIP › Supplementary Figure 1.pdf]
